# Microbiome applications for laying hen performance and egg production

**DOI:** 10.1016/j.psj.2022.101784

**Published:** 2022-02-18

**Authors:** Steven C. Ricke, Dana K. Dittoe, Elena G. Olson

**Affiliations:** Meat Science and Animal Biologics Discovery Program, Department of Animal and Dairy Sciences, University of Wisconsin, Madison, WI 53706, USA

**Keywords:** microbiome, egg production, layer hens, gastrointestinal tract

## Abstract

Management of laying hens has undergone considerable changes in the commercial egg industry. Shifting commercial production from cage-based systems to cage-free has impacted the housing environment and created issues not previously encountered. Sources of microorganisms that become established in the early stages of layer chick development may originate from the hen and depend on the microbial ecology of the reproductive tract. Development of the layer hen GIT microbiota appears to occur in stages as the bird matures. Several factors can impact the development of the layer hen GIT, including pathogens, environment, and feed additives such as antibiotics. In this review, the current status of the laying hen GIT microbial consortia and factors that impact the development and function of these respective microbial populations will be discussed, as well as future research directions.

## INTRODUCTION

Commercial egg production has undergone bumerous changes in the past decades. Several factors have been responsible for these changes. From an economic standpoint, the commercial egg-laying industry has experienced shifts from small farm flocks to much larger egg production operations. This has included the development of egg processing management systems, namely in-line where eggs are collected and processed on-site vs. off-line where eggs are collected then transported to another facility for processing (Musgrove, 2011; Ricke, 2017a). As these operations became more sophisticated and the egg market demand increased, the number of birds per egg-laying flock and birds per layer house increased (Kidd and Anderson, 2019). Much of this expansion in birds per flock and house has been due to management improvements such as mechanical egg gathering apparatus in in-line egg operations (Bell et al., 2001; Kidd and Anderson, 2019). These improvements, along with technological advancements in various steps in egg processing such as candling, egg washing, and packaging, resulted in the increased efficiency of commercial egg production (Kuney et al., 1995; Hutchison et al., 2003; Musgrove, 2011; Ricke, 2017a; Kidd and Anderson, 2019). In addition, scientific, nutritional, and genetic progress in understanding the egg from a chemical, physical, and functional perspective as well as egg characteristics such as shell color, strength, and chemical composition offer further directions for assessing commercial egg quality (Bain, 2005; Rossi et al., 2013; Stoddard et al., 2017; Wilson, 2017).

More recently, the introduction of animal welfare considerations and perceptions has led to further changes in layer hen housing that has impacted production practices in the commercial egg industry (Kidd and Anderson, 2019). These initiatives ultimately led to the cage-free egg layer management operations becoming a part of commercial egg production (Anderson, 2009; Holt et al., 2011; Mench et al., 2011). In turn, this shift in housing management has led to unique challenges in laying hen welfare, health, and performance, such as increased exposure to certain diseases and parasites (Lay et al., 2011; Jeni et al., 2021). While bird physiology is critical to responses to these challenges, the gastrointestinal tract (**GIT**) and its concomitant microbial ecology are also important (Jeni et al., 2021). This importance is not surprising as the laying hen GIT microbial population has been identified as critical for nutritional responses and interaction with the immune system. Historically, the laying hen GIT microbiota has also been associated with foodborne pathogen colonization and infection, particularly *Salmonella* serovars such as *Salmonella* Enteritidis (Ricke, 2017b, 2021). As more has become known about the laying hen GIT microbial ecology, interest has increased for developing strategies to modulate it for the benefit of the bird; however, more needs to be understood about the laying hen GIT microbial population composition and functionality. In addition, the impact of the environmental microbial ecology on egg production and quality may be a contributing factor. In this review, the current status of the laying hen GIT microbial consortia, egg microbiology, and factors that impact the development and function of these respective microbial populations will be discussed, as well as future research directions.

## Potential Sources of the GIT Microbiome in Laying Hens

The relationship between the laying hen and the microbiology of the egg has been a topic of interest for several decades. Traditionally the concern has been on the impact of microbial contamination of table eggshell surfaces, antimicrobial defenses of the egg, and the potential for egg quality deterioration and egg rot (Tranter and Board, 1982; Mayes and Takeballi, 1983). Consequently, efforts to develop egg washing and sanitation procedures for egg processing evolved as more became understood about the microbial ecology of the eggshell surface (Hutchison et al., 2003). The microbial populations on eggshells can vary by geographical region and shift during storage time (Shi et al., 2020). In addition to egg spoilage, the presence of foodborne pathogens on eggshell surfaces remains a public health concern. This contamination is particularly true for *Salmonella*, which can be a risk whether it remains on the eggshell surface or gains access internally (Howard et al., 2012; Galiş et al., 2013; Ricke, 2017b). Now that housing changes have been implemented in the commercial egg industry, the microbial ecology of eggshell surfaces and the opportunity for exposure to *Salmonella* and other pathogens has likely become altered as well (Holt et al., 2011). Understanding the dynamics of the potential changes in environmental microbial ecology in commercial egg production has implications for table egg microbial contamination. It also offers a possible understanding of the origins and potential sources of GIT microbiota in the young layer chick as it emerges from hatching.

The microbial ecology relationship between the laying hen and the developing egg became much clearer when the mechanism of transovarian *Salmonella* infection of the egg and subsequent deposition of the pathogen internally in the egg was established (De Buck et al., 2004; Gantois et al. 2009; Howard et al., 2012; Galiş et al., 2013; Ricke, 2017b). This may be a *Salmonella* serovar-specific response as different serovars potentially possess differing levels of tissue tropisms, with some serovars such as *S.* Enteritidis exhibiting an affinity for layer hen reproductive tract organs (Gantois et al., 2009; Kaldhone et al., 2016; Ricke, 2017b). Once established in the reproductive tract, *S.* Enteritidis can be deposited in the internal egg contents as the egg is being formed. After being internalized, *S.* Enteritidis becomes almost impossible to either detect in the egg or eliminate with externally applied sanitizers. This has resulted in control measures at the retail level that are either focused on minimizing temperature abuse during table egg retail operations, derivation of appropriate cooking temperatures, and decreasing initial establishment of *S.* Enteritidis in the laying hen. Consequently, developing management and nutrition strategies to reduce *Salmonella* colonization in the laying hen GIT during the egg-laying cycle remains important (Holt, 2003; Ricke, 2003a, 2017b). Discovering this alternative route for *Salmonella* contamination of table eggs also suggests that the microbial ecology of the layer hen reproductive tract could impact the ultimate microbial profile of table eggs and the eggs that are to be hatched out as chicks. *In ovo* microbial inoculation studies would also support the opportunity for early exposure and impact on the development of the chick embryo microbiome (Roto et al., 2016; Peebles, 2018). This would also imply a maternal influence on the outcome of the microbial composition of the embryo and the freshly hatched chick. When Ding et al. (2017) compared the GIT microbial composition of embryos, chicks, and maternal hens, they concluded that at least a proportion of the early embryo microbial colonizers originated from the maternal hens.

More recently, Lee et al. (2019) characterized the microbial ecology of the laying hen oviduct and its impact on the chicken embryo microbiota. They sampled the oviduct mucosal surfaces of four euthanized 32-wk-old laying hens, focusing on the infundibulum, magnum, isthmus, and uterus regions to identify the microbial populations inhabiting these regions. In addition, they sampled 25 specific pathogen-free layers before egg-laying and during laying to assess the impact of the environmental reproductive tract microbial communities on the fertilized eggs being deposited. Along with oviducts of selected birds that were euthanized during the study, eggshells and live bird cloacal surfaces were swabbed along with a sampling of fertilized egg whites by syringe. Following incubation, the ceca of 18-day old chick embryos were collected. When different reproductive tract compartments were compared, the microbial taxa were similar throughout the tract, with a relatively high abundance of lactobacilli species detected. Still, they were much lower in the embryo GIT, leading the authors to suggest minimal transfer from the oviduct; however, lactic acid bacteria can become established relatively early in the GIT. Rodrigues et al. (2020) reported that probiotic lactic acid bacteria introduced *in ovo* might serve as pioneer microorganisms in the ileum of young broilers. It would be of interest to determine where poultry GIT lactobacilli originate since they can be isolated throughout the GIT tract of young birds and remain detectable in the crop and GIT of older broilers and laying hens (Durant et al., 1999; Rehman et al., 2007; Dibner et al., 2008; Adhikari and Kwon, 2017).

Lee et al. (2019) noted that the number of microbial species increased in the oviduct as the layer hens matured with greater diversity occurring in the mature hen oviduct compartments compared to their immature counterparts with genus *Pseudomonas, Lactobacillus, Megamonas, Bacteroides*, and *Oscillospira* taxonomically classified as core genera in all mature hen magnums vs. only *Pseudomonas* and *Acinetobacter* in immature hen magnums. When diversity metrics (Principal Coordinate Analyses (**PCoA**) Plots, PERMANOVA, and ANOSIM) were compared among reproductive tract and respective egg microbial populations, Lee et al. (2019) detected similarities between the egg white and the embryo. From these findings, Lee et al. (2019) concluded that the egg white was a primary source of the embryo GIT microbial consortium. Based on ANOSIM comparisons, similarities of the reproductive tract microbial populations and the egg white and embryos were not detected. However, using a SourceTracker2 test, the authors suggested that there was a linkage between the eggshell microbiota and the egg white and embryo microbiota with over 50% potentially originating from the eggshell and in turn, the eggshell receiving microbiota from the maternal cloaca and oviduct with 21 bacterial genera in common for the maternal cloaca, magnum, eggshell, egg white, and embryo. While the authors suggest that the oviduct influences the cloacal microbiota profile, which in turn, impacts the microbial establishment on the eggshell, egg white, and embryo, more research is needed to establish the linkages between these niches. Attempts to use marker strains or genomic barcoded bacterial core oviduct strains, which can be quantitatively tracked, could help delineate the factors that may influence these transmission routes. Once these linkages are better known, modulation of the microbial populations at critical interfaces could be applied to modify the developing microbiota in the embryo GIT and the hatched layer chick to benefit bird health.

## Influential Factors on Sources of the GIT Microbiome in Laying Hens

Based on the potential linkages between maternal hens and their eggs, the eggshell surface would appear to be a critical source of microorganisms for the hatched chick. Maki et al. (2020) investigated the bacterial relationships between the eggshell, environment, and the GIT of the young chick. Fertilized eggs were segregated into three respective incubation conditions to separate the contributions of each microbial source. The incubation conditions consisted of environmental exposure only, eggshell contact only (eggshell microbiota as the only inocula), and a combination of both. After hatching, chicks were placed on a litter, weekly fecal samples were collected, and subsets of chicks were removed on wk 1, 3, and 6 for sampling cecal, ileal, and jejunal luminal contents and conducting mucosal swabs. Eggs were either swabbed or immersed in sterile bags containing a buffered diluent to recover DNA and viable bacteria. Viable anaerobe microbial culturing, SCFA analyses and 16S rDNA sequencing were employed to assess the microbial composition of these respective sources. When following the microbial populations on eggshells during incubation, the numbers of anaerobes initially were high. Still, they declined by d 7 prehatch, and a smaller fraction identified as spore formers remained constant. Based on 16S rDNA sequencing and amplicon sequence variant (**ASV**) taxonomic assignment, some of the more predominant core microbial genera included *Lactobacillus, Enterococcus, Romboutsia*, and *Escherichia*. Based on the sharing of over 50 ASVs among eggshells exposed to different environmental sources and recovery of culturable microorganisms from eggshell surfaces, the authors concluded that these results indicated maternal transmission of maternal microbiota to the hatched chick.

Specific microbial relationships were less clear. For example, the authors could not detect *Lactobacillus* in the upper GIT even though *Lactobacillaceae* were predominant on eggshells which they noted was somewhat surprising Adhikari and Kwon (2017). were able to detect *Lactobacillus* spp. throughout the GIT lumen and mucosa of 3-week broilers, but they used selective de Man, Rogosa, and Sharpe (**MRS**) agar plates for recovery, which would optimize the growth of *Lactobacillus*. However, Maki et al. (2020) also reported that the source of eggshell microbiota did impact the evolution of microbial composition and SCFA profiles on the different GIT segments as the hatched chicks developed. In summary, this further supports the concept that eggshell microbiota may very well be at a crossroads between maternal and hatched chick GIT microbiota development and, as Maki et al. (2020) concluded, represents a potential modulation opportunity for improving GIT health.

Other factors may influence the interaction between the eggshell microbial composition and the ultimate profile of the microbiota transferred to the chick GIT. Trudeau et al. (2020) examined the impact of parental fecal microbiota on the corresponding eggshell microbial composition. The authors collected fresh fecal material and swabbed eggshell surfaces from 12 free-run commercial broiler breeder flocks twice over 4 wk. When the respective fecal and eggshell microbial populations were compared, they concluded that both flock and sampling time differentially impacted both alpha and beta diversities in fecal and eggshell microbial communities. The finding suggests that flock origin, management, and timing of sampling relative to the age of the layer flock could influence the eggshell microbiota and, in turn, the development of the posthatch chick GIT. It would be interesting to follow chick GIT microbiome development in chicks hatched from a young laying flock vs. the same but older flock. These differences could impact various GIT health and performance responses in the birds originating from different ages of breeder hens. Host selectivity could also develop in other ways. With the introduction of transcriptomics to the laying hen ovary and other tissues (Overbey et al., 2021; Zhang et al. 2021), it is may be conceivable in future studies to identify the host defense mechanisms in the laying hen reproductive tract that prevent colonization by certain bacterial groups and, thus, influence microbial community that encounters the eggshell surface.

Trudeau et al. (2020) identified *Lactobacilliaceae* as a predominant family in fecal samples based on 16S rRNA taxonomic identification. However, this bacterial family was not detected on the eggshells along with other characteristic fecal microorganisms. This finding led Trudeau et al. (2020) to suggest that fecal microbiota transfer to the eggshell was not consistent and potential selectivity could be occurring. Other environmental sources may also be responsible for the eggshell microbial composition observed in their study. Observations made by Trudeau et al. (2020) are consistent with the findings by Richards-Rios et al. (2020) on the inability to transplant certain core adult cecal bacteria sprayed onto hatching eggs. Trudeau et al. (2020) suggested air, litter, and dust contained microorganisms identified in previously published studies that could be detected on eggshells implying that these environmental sources may also be contributors. In addition, Trudeau et al. (2020) noted that feathers and skin could also contribute to eggshell microbial ecology. With wild birds, feathers serve as nesting material and are predominant sources of the microbial populations found on the eggshells (Lee et al., 2014; Van Veelen et al., 2018; Trudeau et al., 2020). Feathers may have a more selective impact on eggshell microbial composition as well. Feathers are known to bioaccumulate antimicrobial residues, which can become inhibitory to certain bacteria when feathers are processed into feather meals for animal nutrition (Love et al., 2012). Whether leaching of these bioaccumulated antimicrobials occurs in intact feathers incorporated as nesting materials is unknown. However, in wild birds, feathers used as nest lining material have been demonstrated to inherently produce antimicrobial substances that appear to impact eggshell microbial density (Peralta-Sánchez et al., 2012). Feather pigmentation is also a factor, as Peralta-Sánchez et al. (2014) demonstrated that unpigmented or white feathers yielded higher antimicrobial activity than pigmented feathers. The presence of these antimicrobial compounds could be a part of the selectivity that contributes to maternal transfer to eggshell microbial composition. Consequently, as laying hen egg production moves to more cage-free systems, factors such as more exposure to feathers may play a role in eggshell microbial composition, influencing embryo and chick GIT microbial development.

## Development of the GIT Microbiome in Adult Laying Hens

Several factors that influence the development of the layer hen GIT microbiome differ from the broiler GIT microbiome. A significant factor is the much longer life span of the layer hen vs. the broiler bird (Rychlik, 2020). There are also indications that the breed of either the broiler or the layer chick may influence the early development of the GIT microbiota. For example, when Walugembe et al. (2015) compared male Ross broiler chicks and male Hy-line W36 laying hen chicks fed either low or high fiber diets, they noted differences in the GIT microbial activities of the respective breed of bird. Their results were based on cecal samples collected on d 21 that were analyzed for SCFA, terminal restriction fragment length polymorphisms (**TRFLP**), and metagenomic analyses. Broiler chicks exhibited greater concentrations of acetic and propionic acids than layer hen chicks, and variation in TRFLP cecal microbial profiles occurred due to both fiber level and birds’ breed. Diets containing more significant levels of fiber also impacted the cecal microbiome, with increased abundances of *Campylobacter jejuni, Helicobacter pullorum, Megamonas hypermegale* being noted in broilers and *Escherichia coli* in both broilers and layers. *Helicobacter pullorum* and *Megamonas hypermegale* were also detected in more significant proportions in layer chicks fed low fiber diets. At the same time, *Faecalbacterium* was greater in broilers fed low fiber diets, but the reverse was true in layer chicks. These results suggest GIT microbiota compositional and functional differences occur between layer and broiler chicks even at a relatively early stage. This difference may impact both the rate and overall microbial compositional changes that evolve as the respective bird type matures into adults.

The early colonization and presence of specific pathogens such as *Salmonella* may alter the development of GIT microbiota in young chicks. There is some evidence both with *S.* Enteritidis oral inoculation in young male chicks at different ages and a comparison of *S.* Typhimurium vaccine strains that some impact on the cecal microbiota development postexposure can be detected (Juricova et al., 2013; Videnska et al., 2013a; Park et al., 2017a). This interaction appears to occur in layer chicks as well Mon et al. (2015). orally introduced *S.* Enteritidis to 1-day old layer chicks followed by euthanization at 2 and 7 d posthatch to collect cecal contents for *Salmonella* enumeration and microbiome analyses. Overall, cecal alpha diversity based on Shannon's index increased as the birds matured. ANOSIM analyses for beta diversity revealed significant differences in the microbial populations between the 2 age groups. When infected birds were compared to their uninfected counterparts, the presence of *S.* Enteritidis in the younger birds suppressed cecal microbial alpha diversity and increased the proportion of members of the *Enterobacteriaceae* family, but alpha diversity was similar in the older birds. However, unweighted UniFrac analyses of PCoA plots revealed beta diversity differences in the respective microbial populations between the 2 groups of older birds vs. no differences detected in the younger birds. When taxonomic identification was included, the presence of *S.* Enteritidis was associated with a decrease of members of the *Lachnospiraceae* family Mon et al. (2015). concluded that the introduction of *S.* Enteritidis in young birds could impact the development of the cecal microbial consortia over time. The apparent inverse relationship between *S.* Enteritidis and the *Lachnospiraceae* family suggests potential antagonism between the 2 groups.

Ballou et al. (2016) examined the influence of a *S*. Typhimurium live vaccine and a commercial probiotic combination of *Lactobacillus acidophilus, L. casei, Enterococcus faecium*, and *Bifidobacterium bifidum* on cecal microbial ecology development in layer chicks from the day of hatch until 28 d. Chicks were removed throughout the study on d 0, 1, 3, 7, 14, 28. Following euthanization, cecal contents were removed for microbiome analyses. Based on comparisons of cecal microbiomes as a function of age and treatment, Ballou et al. (2016) concluded that age had more impact than treatment. However, the authors noted that despite the transient properties of both the *Salmonella* vaccine and the probiotics, there were detectable beta diversity impacts on cecal microbiome development on d 1, 7, 14, and 28. The authors detected a rapid change in the cecal microbiome on d 1 and 3, beginning with *Enterobacteriaceae* predominance, followed by an increase in Firmicutes by d 7, with the impact of treatment becoming more prominent as the cecal microbiome developed further. However, when the authors examined the functionality of the cecal microbiome using metagenomic inferences from the QIIME platform, they concluded that the resident cecal microbial population might be more diverse at 14 rather than 28 d. Based on these studies, it appears an opportunistic pathogen such as *Salmonella* can alter cecal microbial development in young layer chicks, even in the form of a single-dose vaccine. The response may be an indicator of the influence of the vaccine on the immune system of the young chick and the collateral impact on the establishment of the cecal microbial population; however, the degree of effect may be related to the GIT resident time of the pathogen. Therefore, both studies with multiple/repeat doses of *Salmonella* vaccine and nonvaccine studies with *Salmonella* residing in the layer chick GIT for more extended periods may need to be conducted to delineate short term vs. long term occupancy influence by *Salmonella* on GIT microbial development. In addition, different serovars may need to be examined to determine if *Salmonella* serovars differ in their interaction with the cecal microbiota.

Different poultry GIT compartments such as the crop may also need to be included to delineate overall GIT microbial responses vs. those focused strictly on the cecum. Given the longer life span, there are opportunities for the laying hen GIT microbiota to continue to evolve beyond the early stages of chick growth and development as the birds become older. Four stages of cecal microbiota development in laying hens have been defined using 16S rDNA sequencing targeting the V3–V4 region, from the day of hatch to 60 wk of age by Videnska et al. (2014). During the first study, diets were void of any antibiotics, and the flock of Lohmann Brown Light layers were vaccinated against coccidiosis on d 10 and reared in a commercial setting (Videnska et al., 2014). The second study was conducted in a controlled research environment (wired battery cages) with male ISA Brown chicks to confirm the first study's results. During the first 3 wk of life at family or higher-level taxa, similar growth characteristics were observed both for commercially reared chickens and chickens raised in a clean experimental animal house with controlled conditions including air conditioning, air filtration, and the absence of contact with rodents or insects (Videnska et al., 2014).

As a result, they identified the first stage of cecal development that occurs immediately after hatching. The ceca become populated by facultative anaerobes of the genus *Escherichia*, family *Enterobacteriaceae* of the phylum Proteobacteria. The second stage (weeks 2 to 4) was characterized by almost complete predominance of members of the Firmicutes phylum, namely *Lachnospiraceae* and *Ruminococcaceae*. The third stage (2 to 6 months) was characterized by the inverse relationship between Firmicutes and Bacteroidetes where the population of Firmicutes increased, and Bacteroidetes decreased. The fourth stage consisting of layers 7 months or more in age, possessed a constant ratio of Bacteroidetes and Firmicutes which the authors considered to be more in line with the characteristic cecal microbiota of what would be associated with adult hens.

The type of housing may impact the development stages of the layer hen GIT microbial communities to some extent (Ricke and Rothrock, Jr., 2020). Some of this may be related to the environmental temperature. Zhu et al. (2019) noted that heat stress could shift fecal microbiome composition and functionality in caged layers; however, Schreuder et al. (2021) did not detect changes in cloacal microbial population composition when they compared layer flocks over 16 weeks that were either given access to outdoors or remained inside. The only shifts occurred in rare taxa and none in the proportionally dominant taxa associated with these birds. Where and when layer hen microbiota is sampled may be a factor as well. Adhikari et al. (2020) collected cecal samples from 2 genetic lines of layer hens at later stages of egg production (53, 58, 67, and 72 wks of age) housed in either conventional cages or enriched colony cages. Depending on the week of sampling, they observed different beta diversities and predicted functionalities in cecal microbial populations between the 2 housing environments. There are some indications that different egg-laying stages may impact cecal microbiota diversity in conventional and cage-free layer hens, even though bird stress markers do not appear to be affected (Van Goor et al., 2020). Cui et al. (2017) compared the small intestines and cecal microbiota of free-range and caged hens digesta collected from young (8 wk) and mature (30 wk) hens. Based on PCR-DGGE and subsequent sequencing of bacterial 16S rDNA gene amplicons, they concluded that age and housing impacted the individual GIT microbial population compositional profiles.

In summary, it appears that efforts will need to be taken to monitor layer hen GIT microbial populations throughout their life cycle in comparative studies between different types of housing. Delineating housing type impact vs. other factors such as environmental temperature, nutrition, and potential contributing external inputs will be needed to develop optimal management strategies for maintaining layer hen health and egg production.

## Microbial Metabolism and Fermentation in the Layer Hen GIT

As the adult layer hen GIT becomes established and more diverse, this can also be reflected in the functional activities of the GIT microbiota. The functionality of the GIT microbial community can be attributed to the combination of substrate hydrolysis/utilization capabilities of the individual microbial members and the resulting generation of metabolite end-products. The generation of metabolites from GIT microbial activities can, in turn, potentially impact the host. For laying hens, this contribution could affect both the physiology of the host and egg production. Further complexity is added to this relationship due to the ability of some GIT microorganisms to possess multiple fermentation pathways, which can shift depending upon factors such as substrate availability. In addition, the predominant microbial populations can vary in different segments of the layer hen GIT, potentially resulting in various fermentation end-product profiles depending on the GIT segment indigenous microbial population. For example, due to the dominance of lactic acid bacteria in the crop, lactate is likely to be a significant product in the crop. However, this dominance can vary as some lactobacilli are heterofermentative and capable of producing SCFA depending on the GIT environmental conditions and substrate availabilities (Russell and Cook, 1995). This phenomenon is also likely true in the upper GIT as *Lactobacillus* spp. vary in the intestinal tract (Adhikari and Kwon, 2017).

The cecum has always been considered the primary site for harboring more predominant anaerobic fermentative microbial populations in the layer hen GIT. This fermentation is evident from the relatively high levels of SCFA produced in the ceca (Józefiak et al., 2004; Ricke, 2020; Ricke and Rothrock, Jr., 2020). Likewise, it appears that layer hen cecal microorganisms can ferment a wide variety of carbohydrate substrates with different fiber compositional profiles to generate SCFA. For example, utilizing an *in vitro* layer hen cecal anaerobic incubation model, Dunkley et al. (2007b) screened a multitude of fiber-containing feed sources, including soybean meal, soybean hull, beet pulp, wheat middlings, ground sorghum, cottonseed meal, alfalfa meal, and alfalfa meal-commercial layer ration mixtures. They concluded that SCFA production only occurred in the presence of the feed source, with virtually no fermentation being detected in cecal inocula alone. However, various feed sources supported different cecal microbial communities when DGGE patterns were used to assess microbial community diversity. The SCFA profiles detected in adult laying hen ceca have been reported to consist primarily of acetate, propionate, and butyrate with much lesser quantities of isobutyrate, isovalerate, and methylbutyrate, among others, along with lactate production (Corrier et al., 1997; Woodford et al., 2005; Dunkley et al., 2007a; Donalson et al., 2008a). Typically, of the three major SCFA, acetate occurs in the highest levels followed by lesser amounts of propionate and butyrate, and these increase over time when layer hen cecal contents are incubated in *in vitro* anaerobic batch culture systems (Dunkley et al., 2007b; Donalson et al., 2008b).

Other fermentation products such as methane and hydrogen are also generated in the layer hen ceca. Both *in vitro* and *in vivo* studies on cecal fermentation have also revealed the presence of methanogens in layer hen cecal contents Saengkerdsub et al. (2006). conducted *in vitro* cecal incubation studies with cecal mixtures mixed and pooled from 56 to 72-wk-old Leghorn hens followed by dilution to a 1:20 concentration with a combination of anaerobic culture medium and anaerobic dilution solution containing sodium formate along with either layer ration or alfalfa fiber. The *in vitro* incubations were carried out for 24 h under a carbon dioxide and hydrogen gas mixture, where SCFA, methane, and hydrogen levels were monitored. Both feed sources supported methane production, with the fiber-based incubations increasing methane production by over 3-fold vs. the layer ratio alone (59.1 vs. 19.1 mmol/g cecal content, respectively). In addition, methanogens could be detected in these cecal contents using 16S rRNA genes and were tentatively identified as *Methanobrevibacter woesei* (Saengkerdsub et al., 2007). Further proof was demonstrated with the addition of methane inhibitors which, for the most part, reduced methane production and, after exposure to the respective inhibitor, shifted to more hydrogen being detected.

Noncarbohydrate end products from fermentative activities associated with nitrogen, sulfur, and protein metabolism also occur in the layer hen ceca. In general, amino acids derived from endogenous and undigested intact dietary protein sources can enter the ceca, deaminated to ammonia and ammonia-containing compounds by cecal microorganisms followed by absorption by the bird (Karasawa, 1989). Dietary urea and urine from the cloaca can also enter the ceca and be converted to ammonia (Karasawa, 1989; Svihus et al., 2013). Specific feed ingredients containing higher levels of choline such as rapeseed or fishmeal-based diets have been attributed to the production of trimethylamines (**TMA**) in the chicken ceca (Hobson-Frohoch et al., 1975; March and MacMillan, 1979; Pearson et al., 1983; Emmanuel et al., 1984; Dänicke et al., 2006). Trimethylamines derived from cecal bacterial metabolism of choline can be a sensory problem in the eggs produced from layer hens being fed these diets due to the "egg taint" that can best be described as a fishy odor that occurs from TMA being deposited in the egg contents if the layer hens' TMA oxidase is overloaded (March and MacMillan, 1972, Pearson et al., 1983; Dänicke et al., 2006).

Hydrogen sulfide has also been identified as an odor compound resulting from cecal microbial activity. Sulfur amino acids may serve as a source of hydrogen sulfide, as evidenced in broiler work where dietary methionine sources have been linked with the presence of hydrogen sulfide in fecal excreta (Chavez et al., 2004). Whether this is related to cecal microbiota is unclear; however, Kumar et al. (2019) examined the influence of supplemental DL-methionine on the cecal microbiota of broilers. They concluded that the presence of dietary methionine impacted cecal bacterial energy and glucose metabolism and increasing dietary methionine in the late stages of the 42-d broiler growth cycle increased glycolysis and energy generation. The presence of the sulfate-reducing bacteria *Desulfovibrio* has also been detected in the cecum of layer hens (Peralta-Sánchez et al., 2019). Huang et al. (2019) used 16S rRNA sequencing and qPCR to compare Hy-Line Gray and Lohmann Pink laying hen breeding lines for potential sources of odor gas emission of hydrogen sulfide production. Based on the taxonomic assessment, sulfate-reducing bacterial genera *Mailhella* and *Lawsonia* aligned with hydrogen sulfide production. At the same time, butyrate-producing bacteria *Butyricicoccus, Butyricimonas*, and *Roseburia* were inversely related, leading the authors to suggest that these 2 metabolic groups were potentially antagonistic with each other. Lower hydrogen sulfide was detected in Hy-Line hens vs. the Lohmann hens. This observation matched the greater proportion of sulfate-reducing cecal bacteria in the Lohmann hens and the results from *in vitro* fermentation studies comparing the cecal inocula from the 2 breeds. Independent confirmation of this outcome was accomplished by quantifying the sulfate reduction functional gene aprA (adenosine-5′-phosphosulfate reductase alpha subunit gene) using qPCR and demonstrating that its relative proportion was also higher in Lohmann layer hens. As the authors concluded, these results provide insight into the contribution of the cecal microbiota to environmental characteristics associated with layer hen management, such as odor emissions, and offer potential means for mitigation. These mitigation strategies may incorporate the choice of breed and potentially modulate the cecal microbiota to a population more antagonistic to sulfide production. However, efforts are needed to characterize the cecal metabolome and metagenomic assessment of genes involved in these pathways to develop more targeted mitigation strategies.

## Imbalances Occurring in the GIT Microbiome of Adult Laying Hens

While the adult laying hen GIT microbial community is generally viewed as being stable after becoming established in the mature bird, disruptive events can occur, resulting in temporal or sustained imbalances in the GIT microbial community. Simon et al. (2016) examined the impact of administering an antibiotic mixture (vancomycin, neomycin, metronidazole, and amphotericin-B) by oral gavage to Lohman Brown layer chicks every 12 h for the first 7 d posthatch along with ampicillin and colistin in the drinking water over 21 d. The first phase (7 d) and the second phase (d 8–21) were defined as the respective antibiotic treatments. For GIT microbial analyses, fecal samples were collected on d 8, 22, 36, and 175 with d 8 viable fecal bacteria being enumerated using a most probable number dilution series in Brain-Heart Infusion broth and extracted microbial DNA from all fecal samples characterized with a Chicken Intestinal Tract Chip phylogenetic array. Total cultivable bacterial populations from d 8 fecal samples were nearly 3,000-fold less than those not receiving antibiotics. Still, microbial diversity based on molecular analyses did not vary between the 2 treatments; however, the authors noted that fecal microbial composition exhibited some characteristic differences with antibiotic-fed birds by d 22. These differences consisted of a proportional increase in the phylum Proteobacteria that would include several pathogens vs. a decrease in Firmicutes, represented by bacterial groups such as lactobacilli. Fecal bacteria from antibiotic-free birds conversely consisted of Firmicutes. Once antibiotics were removed, the fecal microbial composition was similar between the 2 treatment groups. The negative impact of a commercial antibiotic on lactobacilli has also been noted in broilers at 2 wk of age with a return to similar levels as antibiotic-free birds by 6 wk even with continued feeding of the antibiotic (Park et al., 2017b).

The question remains whether antibiotic administration later in the life of the layer hen would impact laying hen microbiota GIT microbial populations. Vindeska et al. (2013b) investigated the impact of single vs. repeated antibiotic administration on the fecal microbiota of egg-laying Lohmann Brown layer hens. For single antibiotic therapy, tetracycline or streptomycin were provided in the drinking water for seven consecutive d to 15-wk-old layers. The fecal samples were collected daily from d 0 before the introduction of antibiotics, followed by sampling on d 1 to 4, 7 to 11, 14, and 16. For repeated doses of antibiotics, 46-wk-old hens were administered the same antibiotics for only 2 d, then removed for 12 d, followed by reintroduction for 2 d. Fecal samples were collected from d 0 to 4, 7 to 11, 14 to 18, and d 18 and 21. Fecal microbial complexity was reduced within 2 d of exposure to the respective antibiotic, regardless of single or repeated exposure to antibiotics. Still, once the antibiotics were removed, the fecal microbial complexity recovery occurred but decreased again when the second dose of antibiotics was administered. When compositional analyses were conducted, *Bifidobacteriales, Bacteroidales, Clostridiales, Desulfovibrionales, Burkholderiales*, and *Campylobacterales* were decreased in the presence of both antibiotics. At the same time, increases in the orders *Enterobacteriales* and *Lactobacillales* were detected in the presence of these same antibiotics. This recovery in fecal GIT diversity would suggest that the older layer hen GIT microbiota is relatively resilient and therefore potentially challenging to modulate once the GIT microbiota has become established in the third and fourth stages of cecal microbial development. However, it would be interesting to determine the impact of continuous antibiotic administration over the entire life cycle of the hen from hatch to determine if the GIT microbiota would adapt over time and achieve similar diversity and taxonomic composition as nonantibiotic fed birds. In addition, using metagenomic profiling to assess the prevalence of antibiotic-resistant genes in the respective antibiotic and antibiotic-free backgrounds could illuminate the connection between the length of exposure to antibiotics vs. the time required for sufficient withdrawal. Since antibiotics can potentially transfer from the laying hen into the egg albumen and yolk during some stages of formation (Donoghue et al., 1997a,b; Donoghue and Hairston, 2000), it would also be of interest if this impacted antibiotic resistant patterns in the GIT embryo microbiota.

The adult layer hen GIT can be disrupted by other factors as well. The classic example is the feed withdrawal regime formerly used to induce molt in active egg-laying hens to cause them to cease egg production. The cessation of egg production allowed for a reproductive rest period to prepare the hens for a second egg-laying cycle. After several days feed was reintroduced, and hens initiated a second egg-laying cycle. However, the removal of feed for several days also led to the increased colonization and systemic infection by *S.* Enteritidis and eventually contamination of eggs produced by the infected hens. Further investigation revealed that feed withdrawal disrupted the layer hen GIT creating the opportunity for *S.* Enteritidis to become established (Ricke 2003a, 2017b). More specifically, lactobacilli in the crop were reduced and lactic acid production decreased, thus reducing an initial GIT barrier to *S*. Enteritidis and increasing the expression of the *Salmonella* virulence gene regulator, *hilA* (Durant et al., 1999). In addition, cecal microbial populations were altered, and SCFA production was diminished, leading to a cecal environment favorable to *S.* Enteritidis. Efforts to provide molt diets that retained layer hen GIT microbial fermentation were explored, and some of these not only successively supported layer hen GIT microbial populations and their respective fermentation profiles, but inhibited *S.* Enteritidis establishment (Park et al., 2004; Ricke et al., 2013; Ricke, 2017b).

The laying hen GIT microbial population can be disrupted by *Salmonella* infection even if not undergoing feed withdrawal. It also appears that other *Salmonella* serovars other than *S.* Enteritidis can become established in the laying hen GIT and, in turn, impact the GIT microbiota. For example, Khan and Choukalser (2020) examined the impact of *S*. Typhimurium in the presence or absence of *Bacillus* probiotics (*B. subtilis* or *B. amyloliquefaciens*). They established treatment groups that compared a series of controls, including a negative control, intermittent probiotic control (alternating being fed for four weeks then four weeks not being fed), and continuous probiotic supplementation with the corresponding *S*. Typhimurium challenge (at 18 wk) treatment counterparts. Fecal samples for *S*. Typhimurium quantitation, SCFA, and microbiome analyses were collected on d 3, 5, and 7, followed by inoculation with *S.* Typhimurium on weeks 2, 4, 6, 8, 10, and 12. The collected internal organs, eggshells, and internal egg contents were assessed for *S.* Typhimurium contamination. In general, continuous feeding of the probiotic decreased *S*. Typhimurium levels over time in the feces more than when the probiotic was introduced intermittently. The authors also concluded that continuous probiotic feeding reduced levels in the organs; however, *S.* Typhimurium levels in organs and eggs were generally much lower, being only sporadically detected in some organs, as well as inconsistencies occurring between fecal and cecal levels.

When in-depth taxonomic analyses were conducted by Khan and Choukalser (2020), the presence of *S.* Typhimurium appeared to shift the GIT microbiota based on fecal samples to more genera that would be considered characteristic of dysbiosis. The shift was also observed with the repression of genera traditionally identified as beneficial GIT bacteria such as *Lactobacillus* and *Bifidobacterium*. Different profiles in fecal SCFA were also noted. Butyrate increased in the presence of the probiotic and propionate increased in *S.* Typhimurium-infected layers when probiotics were included. The relationship between probiotics decreasing *Salmonella* and increasing propionate has also been noted in broilers (Nisbet et al., 1996a,b; Ricke, 2003b). Their findings suggest that this particular SCFA, or the microorganisms producing it, may antagonize *Salmonella* in broiler chicks (Nisbet et al., 1996a,b; Ricke, 2003b). Whether this antagonistic relationship in the layer hens is directly due to the increased propionate or indirectly to the competitiveness of the propionate-generating GIT microbiota remains to be determined. Likewise, studying the GIT microbiota composition in these separate compartments could reveal both where the presence of probiotics is most effective in altering the GIT microbiota and where most of the impact on *S*. Typhimurium colonization may be occurring.

## Modulation of the Layer Hen GIT Microbiome

As more sequencing and further characterization studies are conducted on the layer GIT microbiota, opportunities to better understand the relationship between the GIT microbial composition and layer hen egg production performance have emerged. To assess the relationship between egg production and the layer hen GIT microbiota, Wang et al. (2020) segregated Hy-Line Brown hens into high yield birds that exhibited egg-laying rates greater than 96% of the group and low yield hens that possessed egg-laying rates less than 50% of the group. They collected fecal samples from these respective layer hen groups for microbiome sequencing and analyses. In addition, Wang et al. (2020) incorporated fecal microbiota transplantation where layer hens previously grouped as high or low yield egg layers served as microbiome donors for their counterpart after administering amoxicillin for 3 d, followed by oral gavage of the respective fecal suspension twice per day and subsequent fecal sampling for microbiome analyses. When alpha diversity profiles of the 2 fecal microbiota populations were compared, the high egg-laying group exhibited a greater abundance of microorganisms than the low yield birds, with Firmicutes being the dominant phyla followed by Bacteroides for both groups. However, phyla Bacteroides, Actinobacteria, and Proteobacteria were proportionally higher in low egg-yielding birds vs. those detected in the high-yielding birds. At the same time, *Lactobacillus* were relatively more abundant in the high egg-laying hens at the genus level. When egg production and fecal microbiota of the fecal transplant birds were compared after transplantation, the egg production of the high yield layers initially declined but recovered. In contrast, egg production was increased in some of the low-yield birds. This difference was reflected in the microbiota comparisons where the alpha diversity of fecal microbiota from high yield birds initially declined when receiving fecal transplants from low yield hens then rebounded while the alpha diversity in the low yield birds increased after receiving the high yield bird fecal transplants. Wang et al. (2020) concluded that the GIT microbiota of high egg-producing layer hens might be somewhat more stable than those of low-yield birds. This finding would suggest that the perhaps low egg production layer GIT microbial populations are more malleable, and increased egg production could be accomplished via GIT microbial modification.

Attempts have been made to modulate the layer hen GIT microbiota with feed additives. Pineda-Quiroga et al. (2019) compared the cecal microbial responses of 57-wk layer hens fed for 70 d either a prebiotic dry whey powder or the probiotic *Pediococcus acidilactici*, or the combination (synbiotic). When respective cecal microbial populations of the birds sacrificed at the end of the trial were compared, the prebiotic and synbiotic additives led to distinct microbial compositional profiles compared to the control birds. Still, the cecal microbial populations of the birds fed diets supplemented with the probiotic were not different from the control hens. These results were somewhat reflected in the metagenomic analyses. The authors reported that while core metabolic functions were generally not affected, some specific functions were modulated. For example, all feed additives enhanced expression levels of cecal microbial genes involved with starch, sucrose, pyruvate, and glycerophospholipid metabolism. However, prebiotic supplementation also specifically increased butanoate and propanoate metabolism gene expression leading them to suggest that such increases in these SCFA could lead to more metabolizable energy for the host and perhaps partially explain the improved egg production seen in their previous study with this particular prebiotic (Pineda-Quiroga et al., 2017). Such specific alterations in gene expression without influencing overall metabolic activities may fit with the heterofermentative capabilities of certain cecal microorganisms and thus reflect changes in substrates from the prebiotic. This finding is supported by the increase in galactose metabolism related to lactose utilization, presumably from the whey prebiotic fed to the birds. The authors concluded that the more minimal impact of the probiotic might be related to the fact that it was supplemented to older birds; however, the fact that the synbiotic did exhibit a more significant impact indicates that some selection may still be possible if sufficient substrates are available.

There have been efforts to introduce probiotics to laying hens earlier in the production cycle. Peralta-Sánchez et al. (2019) fed the probiotic *Enterococcus faecalis* to 16-week old layers and followed egg production until d 76. Fecal samples were collected on d 7, 15, 40, and 76, and subsets of hens were euthanized on d 40 and 76 for ileum and cecal samples to conduct microbiome sequencing. Once collected, fecal samples were cultivated for indirect detection of *E. faecalis* and anaerobic bacterial enumeration. Sustained egg production throughout the trial was observed when supplementation with probiotics was used. In contrast, the egg production declined in the control birds during the second phase of the trial, after d 40. While the fecal anaerobic enumerated populations did not follow treatments, *Enterococcus* spp. were dominant in the fecal microbial populations, and indirect quantitation of the probiotic *Enterococcus* strain demonstrated an increase in the probiotic bird fecal samples to the point of eventually dominating the fecal microbial population. The microbiome changes in the ileum appeared to reflect the treatment differences as ileum operational taxonomic unit richness appeared to be greater at d 40 for the probiotic-fed birds than control birds but were comparable by d 76. Alpha diversity in the cecum was more diverse than the ileum but was similar between treatments. Still, beta diversity analyses indicated that treatment accounted for most of the microbial variance observed in the cecum. Based on these results, administering probiotics earlier in the egg-laying cycle would appear to have some advantage in egg production; however, this may depend highly on the probiotic organism and its compatibility with the poultry GIT environment. In addition, the probiotic *Enterococcus* in this study was encapsulated in beta-cyclodextrin before adding to the feed after this carrier had been proven to retain cell viability, illustrating the importance of practical delivery systems for poultry production.

Feed additives have been examined for breeder hen performance and GIT microbial responses as well. Wang et al. (2021) were interested in the impact of butyrate from different sources on breeder yellow-feathered hens by comparing low (**CBL**) and high levels (**CBH**) of the butyrate-producing probiotic, *Clostridium butyricum*, with organic acid additives, sodium butyrate (**SB**), and butyric acid glycerides (**BAG**). Breeder hens were given the respective treatments from week 45 to week 54, and eggs were collected during that time followed by incubation in a hatchery, and monitoring of reproductive performance, egg quality, intestinal health, and offspring performance. The treatments impacted egg production and hatched chick performance as both levels of *C. butyricum* and BAG improved the egg-laying rate. However, only the CBH and BAG additives significantly increased daily egg mass and weight while decreasing the feed-to-egg ratio of yellow-feathered breeder hens. Both levels of *C. butyricum* increased albumen height, but only the high level of probiotic increased eggshell thickness and oviduct length. At the same time, only BAG enhanced egg yolk color and increased Haugh unit. All treatments increased the number of large yellow follicles (diameter greater than 8 mm), which is noteworthy since these likely belong to the most mature class of follicles (Howard et al., 2012) and may account for some of the improved egg laying rate seen in some of the treatments.

In the breeder hen GIT, Wang et al. (2021) reported that the high level of *C. butyricum* enhanced expression of several jejunum nutrient transporters and increased jejunum villus height, crypt depth, and both *C. butyricum* levels increased villous/crypt ratio vs. the control birds. The high level of *C. butyricum* and the forms of butyrate also decreased IL-6 in the jejunal contents. Low levels of *C. butyricum* and BAG enhanced villous height, while BAG increased crypt depth. When the cecal microbiota was examined, alpha and beta diversity impacts were less apparent among treatments. However, CBL tended to lower the Shannon alpha diversity index and the total number of detected species vs. the other treatments. When taxonomic identification was conducted, both *C. butyricum* inoculation levels increased the abundance of phylum Firmicutes. However, only the low level increased the abundance of genus *Bacillus* and neither impacted genus *Clostridium*. Since linear discriminant analysis revealed increased abundance of *Shuttleworthia, Lactobacillus, Barnesiellaceae*, and *Bacteroides* for all treatments, the presence of *Lactobacillus* might suggest alterations in GIT pH due to treatment. Still, pH levels were not different in the duodenal, jejunal, and ileal contents of the treated birds compared to the control birds.

When these results are collectively appraised, the impact of the *Clostridium* on the hen GIT is reflected in the hen GIT physiology and, ultimately, some of the egg performance parameters. The butyrate additions exhibited a less clear impact, particularly on the cecal microbiota. The lack of effects may not be surprising as organic acids have been shown to have minimal effects on broiler cecal microbiota responses (Oakley et al., 2014). It would be of interest to examine the GIT microbiota responses in the upper intestinal tract of these breeder hens since several of the nutrient transporters were impacted by the presence of these treatments and determine if certain intestinal microbial groups have overlapping or competing nutrient requirements. Likewise, following the development of the hatched chicks from these breeder hens fed different treatments may reveal whether any influence of diet on the GIT microbiota of the offspring occurs.

## CONCLUSIONS AND FUTURE DIRECTIONS

Substantial progress has been made in developing a more in-depth understanding of the layer hen GIT microbiota. With the introduction of next-generation sequencing for 16S rDNA microbiome characterization and advances in bioinformatics, considerable information is now becoming available to develop practical applications. However, delineating which factors influence GIT microbiota development remains a challenge. The challenge is partly due to the additional environmental complexities introduced by housing management changes from caged birds to a wide range of more open housing versions, such as enriched cages, aviaries, and free-range systems. How much these different housing environments influence initial layer chick GIT colonization through development to an adult layer is not entirely resolved. It is becoming apparent that there is a potential influence on the GIT microbiota development to the point of the initial formation of the egg. Consequently, this presents opportunities for manipulation relatively early in the life of a layer chick, but how best to accomplish this remains to be determined.

Most microbiome studies have focused on the cecum and the representative fermentative microbial population interaction with specific feed additives such as probiotics and different diets. Less effort has been focused on the upper GIT microbial population of the layer hen; however, from a nutritional perspective, the interaction between the upper GIT microbiota and layer hen digestion and absorption could play an important role for bird performance and egg production. For example, increasing fiber levels in layer hen diets have been well documented to alter cecal fermentation. However, recent evidence suggests that there may be an impact on the upper GIT microbiota. For example, when Zheng et al. (2019) examined supplementation of alfalfa meal to female Beijing-you chickens, they observed a trend toward increased *Lactobacillus*, not only in the cecum but the duodenum and ileum as well. However, other fiber sources such as wheat bran have shown a minimal impact on the layer hen GIT microbiota (Wanzenböck et al., 2020). Some brans are known to exhibit prebiotic properties in the presence of cecal microorganisms, but this may depend on the type of cereal grain (Ricke, 2018).

In general, more layer hen studies need to be done with these various fibers and cereal grain bran-supplemented diets to determine which compositional characteristics are most likely to influence layer hen GIT microbial population composition and functionality. Finally, the entire GIT needs to be considered as upper compartments such as the crop may play a more significant role with feed entering the layer hen GIT. Upper compartments may also influence the passage rate, which could have downstream effects on the retention and fermentation of these diets (Ricke, 2018). With the introduction of various proteomic, transcriptomic, and metabolic approaches, the opportunity to dissect layer hen host-GIT microbiota interactions with diet variations should provide insight into optimizing hen GIT health and egg performance parameters.

## DISCLOSURES

The authors declare no conflict of interests were involved in the development of this manuscript.
